# Topographic patterns in the phylogenetic structure of temperate forests on steep mountainous terrain

**DOI:** 10.1093/aobpla/plv134

**Published:** 2015-11-24

**Authors:** Ryo Kitagawa, Makiko Mimura, Akira S. Mori, Akiko Sakai

**Affiliations:** 1 Forestry and Forest Products Research Institute, 1 Matsunosato, Tsukuba, Ibaraki 305-8687, Japan; 2 Department of BioEnvironmental Sciences, Tamagawa University, 6-1-1 Tamagawa gakuen, Machida, Tokyo 194-8610, Japan; 3 Environment and Information Sciences, Yokohama National University, 79-1 Tokiwadai, Hodogaya Ward, Yokohama 240-8501, Japan

**Keywords:** Community assembly rule, heterogeneous environmental condition, phylogenetic diversity, temperate forest in East Asia, topographic gradients

## Abstract

In rugged terrain subject to active geomorphological processes, the species composition of forest communities changes along topographic gradients over short distances. However, the phylogenetic structure of forests on rugged terrain has rarely been examined. Understanding such structures provides insight into community assembly rules dependent on local environmental conditions. To this end, we tested the topographic trends of measurements of phylogenetic community structure [net relatedness index (NRI) and nearest taxon index] in a catchment covered by temperate forests with complex relief in Japan. We found that phylogenetic structure changed from over-dispersion to clustering with increasing slope inclination, change of slope aspect from south to north and decreasing soil depth. This result suggested that environmental filtering tended to restrict community composition at relatively stressful sites, whereas species interaction functioned more strongly at relatively stress-free sites. Relatively stressful sites were characterized by early-successional species that tended to assemble in certain phylogenetic clades, whereas highly competitive late-successional species associated with lower NRI at relatively stress-free sites. However, despite this significant topographic tendency, phylogenetic community structures were not statistically different from random assumptions in most plots. This obscuration of the phylogenetic structures at the plot level could be interpreted as species adapting to disturbances because they were abundant in this catchment. Accordingly, we suggest that a stochastic process was also important for this community at the plot level, although biotic and abiotic environmental filtering controlled the vegetation structure in the catchment, where disturbances occur frequently because of geomorphological mountain denudation processes owing to active crustal movements and abundant rainfall.

## Abstract (precis)

In this study, we tested the topographic trends of measurements of phylogenetic community structure in a catchment covered by temperate forests with complex relief in Japan. We found that phylogenetic structure changed with increasing slope inclination, change of slope aspect from south to north, and decreasing soil depth. Our results suggested that environmental filtering caused by steep topography tended to restrict phylogenetic community composition at relatively stressful sites. On the other hand, species interaction functioned more strongly at relatively stress-free sites.

## Introduction

Understanding the causal factors of fine-grained spatial variations in species diversity and composition within a forest is one of traditional tasks of ecology (Clark *et al*. 1998). Theory suggests that both deterministic and stochastic processes explain spatial variation in community patterns (e.g. Kraft *et al*. 2008; Chase 2010). The deterministic process is based on the classical niche theory, i.e. each species distributes to its own niche, and thus, the members of a community should be determined depending on local biotic and abiotic environment conditions (Hutchinson 1959). On the other hand, the stochastic process has been emphasized in recent studies, involving increases in the randomness of species composition (e.g. Chase 2003). Namely, the occurrence of a species adapting to a local condition at a given site is not assured but is with a certain level of probability caused by factors such as dispersal limitation and/or differential dynamics between colonization and local extinction (Hubbell 2001). Many studies have tried to determine whether deterministic or stochastic processes predominate. Moreover, if a deterministic process is noticeable, the interests of community ecology moves on to reveal the causal factors of the non-random community structure, such as biotic or abiotic environmental factors. Many studies suggest that recent trait-based and phylogenetic approaches are effective for disentangling these questions relating to forest community structure (HilleRisLambers *et al*. 2012). In particular, even in the absence of trait data, a phylogenetic approach could provide insight into the forest community assembly of local habitats (e.g. Kembel and Hubbell 2006; Swenson and Enquist 2009; Kraft and Ackerly 2010; Duarte 2011, Pei *et al*. 2011).

The phylogenetic approach to validate the community assembly has basically evolved from the traditional theory of niche evolution; closely related species can be considered to have similar niches (Harvey and Pagel 1991). Under this assumption, phylogenetic relatedness can be regarded as an index of the differences in niche-related traits, which have come about through evolution. Here, there are two patterns of non-random phylogenetic community structures that are detected by comparison with a random expectation, interpreted as the consequences of two different assembly rules (Webb 2000; Webb *et al*. 2002). Firstly, ‘phylogenetic clustering’, i.e. closely related species co-occur within a community more frequently than expected from a random assumption [lower phylogenetic diversity (PD) than expected], which is interpreted to result from constraints on community members caused by abiotic environmental conditions. Some studies have shown that phylogenetic clustering appears in stressful conditions such as highly disturbed sites (González-Caro *et al*. 2014), and nutrient-poor and arid habitats (Kembel and Hubbell 2006; Spasojevic and Suding 2012). Secondly, if distantly related species tend to co-occur more than expected by chance, this is termed ‘phylogenetic over-dispersion’. This pattern is explained by the rule of limiting similarity, where competitive exclusion acts more strongly between closely related species (Cavender-Bares *et al*. 2004), and/or with facilitation (Verdú *et al*. 2009), which is more probable between distantly related species. Phylogenetic over-dispersion has been detected at sites with relatively mild conditions, e.g. nutrient- and water-rich habitats (Cavender-Bares *et al*. 2004; Spasojevic and Suding 2012), less-disturbed sites (González-Caro *et al*. 2014), and at lower elevations and warm-temperate sites (Qian *et al*. 2014). If neither pattern is detected, the community is interpreted as a phylogenetically random assembly from the species pool (Webb *et al*. 2002; Mouquet *et al*. 2012), or birth filtering and competition are acting in concert. Although the cause-and-effect relationship between these patterns in phylogenetic structure and assembly rule is still under debate, and various interpretations are argued (for example Mayfield and Levine 2010 suggested ‘competition drives phylogenetic clustering’), this conceptual idea is recognized as the basic framework of phylogenetic community ecology.

Therefore, a phylogenetic approach is useful to understand the driving force of spatial variation of species diversity and composition (e.g. Kembel and Hubbell 2006; Spasojevic and Suding 2012; De Oliveira *et al*. 2014; Qian *et al*. 2014). These studies have focussed on habitat heterogeneity, and the phylogenetic structures of local wood communities were examined in relation to environmental conditions, for example as represented by topography. Except for a few studies, this approach has been adopted for tropical and subtropical forests, where there are regarded as being too many species to estimate the assembly rule by detecting niche-related traits of respective species. However, other ecosystems, including temperate forests, have rarely been examined.

East Asian temperate forests show high regional and local plant species richness owing to geological and climatic conditions (Qian and Ricklefs 2000; Qian *et al*. 2005), i.e. complex relief is common because of rapid uplift and abundant rainfall, providing high niche diversity over various spatial scales. In such regions, large variations in species distribution pattern in terms of topography have been reported (Masaki *et al*. 1992; Sakai and Ohsawa 1994; Nagamatsu and Miura 1997; Kitagawa *et al*. 2014). However, local community assembly rules have not yet been investigated in such regions.

Kitagawa *et al*. (2014) analysed the spatial pattern of forest structure for a 306-ha water catchment in a Japanese mountainous area, and they pointed out that local above-ground biomass could be explained through the topographical features of the site. Above-ground biomass was larger around ridges but smaller near valleys, coinciding with an assumable gradient of disturbance intensity from weak on ridges to strong in valleys, which is a pattern that has been reported previously in East Asia (e.g. Sakai and Ohsawa 1994; Enoki 2003; Chang *et al*. 2012). Relating to previous topography–forest structure studies, a hypothesis for community assembly on a topographic gradient can be formulated; if the biomass reflects environmental stress, phylogenetic community structure may change from over-dispersion to clustering with gradient from ridges to valley. However, other topographical variables such as slope aspect, slope inclination and soil depth may also be crucial. Moreover, the function of community assembly in areas with frequent ground-surface disturbance is unclear. Relating to this uncertainty, we assumed that the community assembly rule may be rather stochastic in such highly disturbed conditions (Fukami and Nakajima 2011), whereas it might act as an environmental filter producing more phylogenetic clustering, given the existence of unique disturbance-tolerant species on lower slopes in East Asia (Sakai *et al*. 1995; Qian *et al*. 2005). Verification of these predictions can increase the certainty of the hypothesis for community assembly rules described above and contribute to understand the mechanisms relating to topographical variation of species composition within forests.

Although the above predictions are basically testable by phylogenetic approaches with our inventory data, we should not ignore the uncertainties for the general framework of the phylogenetic approach that were suggested in recent studies (e.g. Godoy *et al*. 2014), in addition to the pattern of phylogenetic niche lability (Losos 2008). In particular, recent studies suggested scepticism about the interpretation of phylogenetic over-dispersion in the existing framework, because they found that competitive interaction was not necessary to limit the phylogenetic similarity within a community. Consequently, we paid attention to species characteristics that contribute to each phylogenetic structure (over-dispersion and clustering). Fortunately, there are many descriptive works on the distribution and ecological characteristics of woody species that are related to local topography in temperate forests in East Asia (e.g. Masaki *et al*. 1992; Sakai and Ohsawa 1994; Nagamatsu and Miura 1997), including our previous study (Kitagawa *et al*. 2014). Accumulation of such information may empirically support the interpretation of phylogenetic community structure, and may complement the concern about the existing framework.

This study aims to understand the community assembly rule along local topographical gradients in a heterogonous mountain landscape. To this end, we examined the topographic trends of phylogenetic structure of 99 study plots within a 306-ha water catchment using the same inventory data as Kitagawa *et al*. (2014), and discuss the distribution pattern of key species groups that affected the phylogenetic structure.

## Methods

### Study area

**Figure 1.**
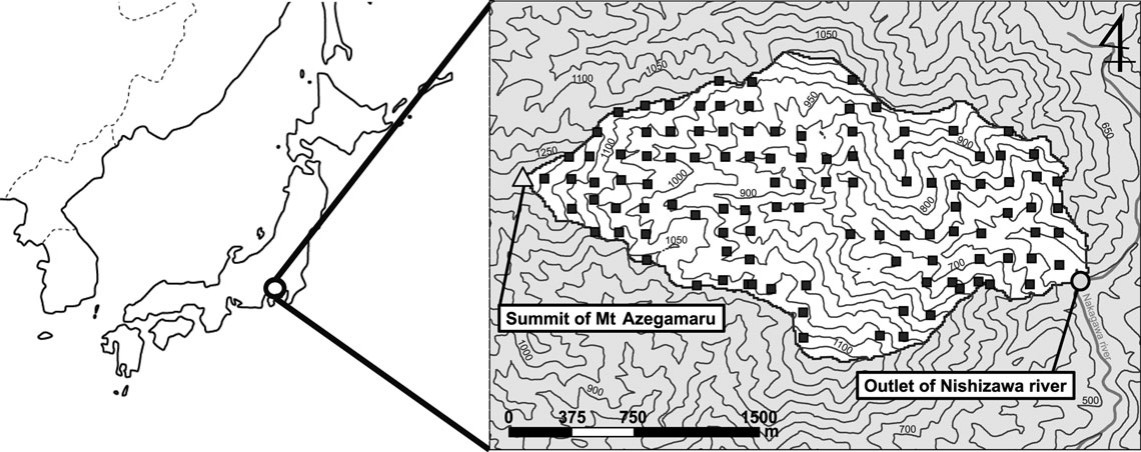
Location of the study catchment and sampling plots, denoted by black squares.

The study catchment (306 ha) is located in the western Mt Tanzawa region, Kanagawa prefecture, central Japan (35°28′00″N–35°29′10″N, 139°01′55″E–139°03′50″E) (Fig. 1). In this study, we targeted one catchment that is a unit of a watershed ecosystem to reveal the overall trends in the community assembly that may depend on the hydrogeological cycle through disturbances relating to mass movements. Mean annual temperature is 12.8 °C (mean temperature in the coldest and warmest months of the year are 2.5 and 23.7 °C, respectively) and the mean annual precipitation is 2819.1 mm, which were all measured at Gotenba (35°18′18″N, 138°55′37.2″E), 472 m above sea level, near the study catchment (AMeDAS by Japan Metrological Agency). The highest point of the catchment is the summit of Mt Azegamaru (1200 m), and the lowest point is the outlet of the main stream, Nishizawa river (560 m). The upper part of the catchment, approximately over 800 m, is covered by cool-temperate forests dominated by deciduous trees, and the lower part is warm-temperate forest dominated by evergreen and deciduous broadleaved trees. The complex terrain of this region consists of steep slopes, which are caused by abundant rainfall, erodible substratum rocks and active crustal movements (Ishikawa *et al*. 2006).

### Inventory data and topographical variables

Ninety-nine plots out of the original 105 plots were used in this study; six plots were excluded because tree species were absent or soil depth data were lacking. To detect the whole trends in community structure in the catchment, these plots were essentially set at the intersection points of every 5 s of latitude and longitude, excluding inaccessible steep slopes and conifer plantations but with the addition of plots on main ridges of the catchment boundary (Fig. 1). To detect geomorphological responses in vegetation, we adopted relatively small plot sizes (10 × 10 m), and slightly moved some locations, because species composition and habitat conditions changed dramatically over short distances in this steep terrain. Species records were for all plant stems that were 5 cm or greater in diameter at breast height (DBH). With the exception of climbing plants, data for all 75 species were used in this study. Mean soil depth was calculated from three recordings in each plot. An iron rod of 50 cm length was used to measure the soil depth, and if the depth was >50 cm, 60 cm was used for analyses.

Slope inclination, laplacian, i.e. an index of concavity and convexity of the ground, elevation and slope aspect were obtained from a 20 × 20 m digital elevation model using Arc GIS (version 9.3, Esri Inc.). Laplacian was calculated using:

$$\frac{nx-\sum_{i=1}^{n}y}{n}$$

where *x*, *y* and *n* are the elevation of the target cell, the elevation of a surrounding cell and the number of surrounding cells, respectively. Positive and negative values show a convex site, such as ridge top, and a concave site, respectively. Each cell was a square of 20 × 20 m. We considered two kinds of laplacian in this study: laplacian using eight surrounding cells (lap8_3; total area 60 × 60 m), which indicated small-scale convexity or concavity of the terrain, and laplacian using 360 surrounding cells (lap8_19; 380 × 380 m), indicating a large ridge or valley that roughly reflected main ridges and valleys of the catchment. Tangent transformation was applied for slope inclination (tan(slope)), and sine transformation was applied for slope aspect (sin(aspect)), ranging from −1 (south facing) to 1 (north facing).

### Phylogenetic structure

To measure phylogenetic distances between species, we constructed a phylogenetic tree: the phylogenies of all species examined in this study were constructed based on the R20120829 megatree in Phylomatic v3 (http://phylodiversity.net/phylomatic/). Branch lengths of angiosperms were assigned based on divergence times estimated by Wikström *et al*. (2001) using the bladj algorithm in PYLOCOM 4.2 (Webb *et al*. 2008). For the calibration of the branches of gymnosperms, we used four calibration points estimated with molecular data and fossil constraints by Magallón *et al*. (2013), including the branching point of angiosperms and gymnosperms [312 million years ago (mya)], the crown age of Pinales (278 mya), the branching point of Taxaceae and Cupressaceae (175 mya) and the crown age of Pinaceae (153 mya).

To evaluate the phylogenetic structure of each plot, we calculated Faith's PD (Faith 1992), the net relatedness index (NRI; Webb 2000; Webb *et al*. 2002) and the nearest taxon index (NTI; Webb *et al*. 2002) using the picante libraries (Kembel *et al*. 2010) in R (http://www.R-project.org; R Development Core Team). Net relatedness index and NTI were defined as:

$$\begin{array}{c} \text{NRI}=-\frac{\text{MPD}-\text{MEAN}(\text{MPDnull})}{\text{SD}(\text{MPDnull})}\\ \;\;\text{NTI}=-\frac{\text{MNTD}-\text{MEAN}(\text{MNTDnull})}{\text{SD}(\text{MNTDnull})} \end{array}$$

where MPD is the mean of pairwise phylogenetic distance between all species in a plot, MNTD is the mean for all species within the plot in relation to the phylogenetic distance from another closest species in the plot, MEAN(MPDnull) and MEAN(MNTDnull) are the means of MPD and MNTD calculated from 999 null communities and SD(MPDnull) and SD(MNTDnull) are their standard deviations. An independent swap approach (Gotelli and Entsminger 2003) was used to generate the 999 null communities, i.e. for each plot, the same number of species as in the plot was chosen at random from the whole species inventory, where the probability of choice for a species was adjusted to the frequency of species occurrence across all plots. Net relatedness index reflects the more general structure, whereas NTI is a more sensitive indicator of phylogenetic extendability or segregation among components.

Positive NRI and NTI values indicated a trend that the community consisted of more-closely related species than expected at random, known as ‘phylogenetic clustering’. On the other hand, negative NRI and NTI values indicated that the community composition was more phylogenetically divergent than expected at random, i.e. ‘phylogenetic over-dispersion’. Here, if the regional flora involves species belonging to much older clades than the majority, the local trends in these indices may be largely affected by the distribution pattern of such older species. Specifically, because the five conifer species might have strong effect on our results, a data set with conifers removed was also used for the calculation of the NRI and NTI. To detect whether a non-random or a random structure was dominant, the numbers of plots that deviated significantly from the random assumption were identified.

### Analyses of topographical trends in phylogenetic structure

To assess the topographic variables meaningful for phylogenetic community structure, linear models were constructed:

y∼elevation+tan(slope)+sin(aspect)+soil depth+lap8_3+lap8_19

as the full model. Response variable (*y*) was any of four indices of the phylogenetic structure (NRI and NTI for all species and the same for only angiosperms), Gaussian distribution was assumed for response variables and linear regressions were fitted. For each response variable, after constructing models for all combinations of the topographical variables, i.e. 144 patterns, we identified the model with the smallest Akaike information criterion (AIC) as the best model, and also detected the other top three models. For these models, *r*^2^ values were calculated to confirm the predictive ability. Spatial autocorrelation for all response variables was not detected by Moran's *I* (NRI for all species: −0.040; NTI for all species: −0.043; NRI for angiosperms: −0.043 and NTI for angiosperms: −0.039). We confirmed that multicollinearity was not detected using variance inflation factors (Fox and Monette 1992) between any explanatory variables except for between two laplacians. Two laplacians did not appear simultaneously in the best model or the other selected models. Each single relationship for the response variables against topographical variables that was selected in the best models of NRI and NTI for all species was tested using linear regression. Because the analysis without conifers was applied to confirm the effect of much older clades for whole trends, single correlations for the selected variables in the best models for NRI and NTI without conifers were not tested.

To understand the contribution of respective species to the observed phylogenetic structures, for each of the 28 species that occurred in 10 or more plots, the frequency of occurrence was calculated for three NRI groups by plot, using NRI for all species, i.e. 33 plots with lower, middle and higher values of NRI, respectively; these NRI ranges were −3.42 to −0.38 for the lower NRI group, −0.29 to 0.66 for the middle group and 0.73–1.81 for the higher group.

All statistical tests were performed in R version 3. 1. 1 (http://www.R-project.org; R Development Core Team).

## Results

**Figure 2.**
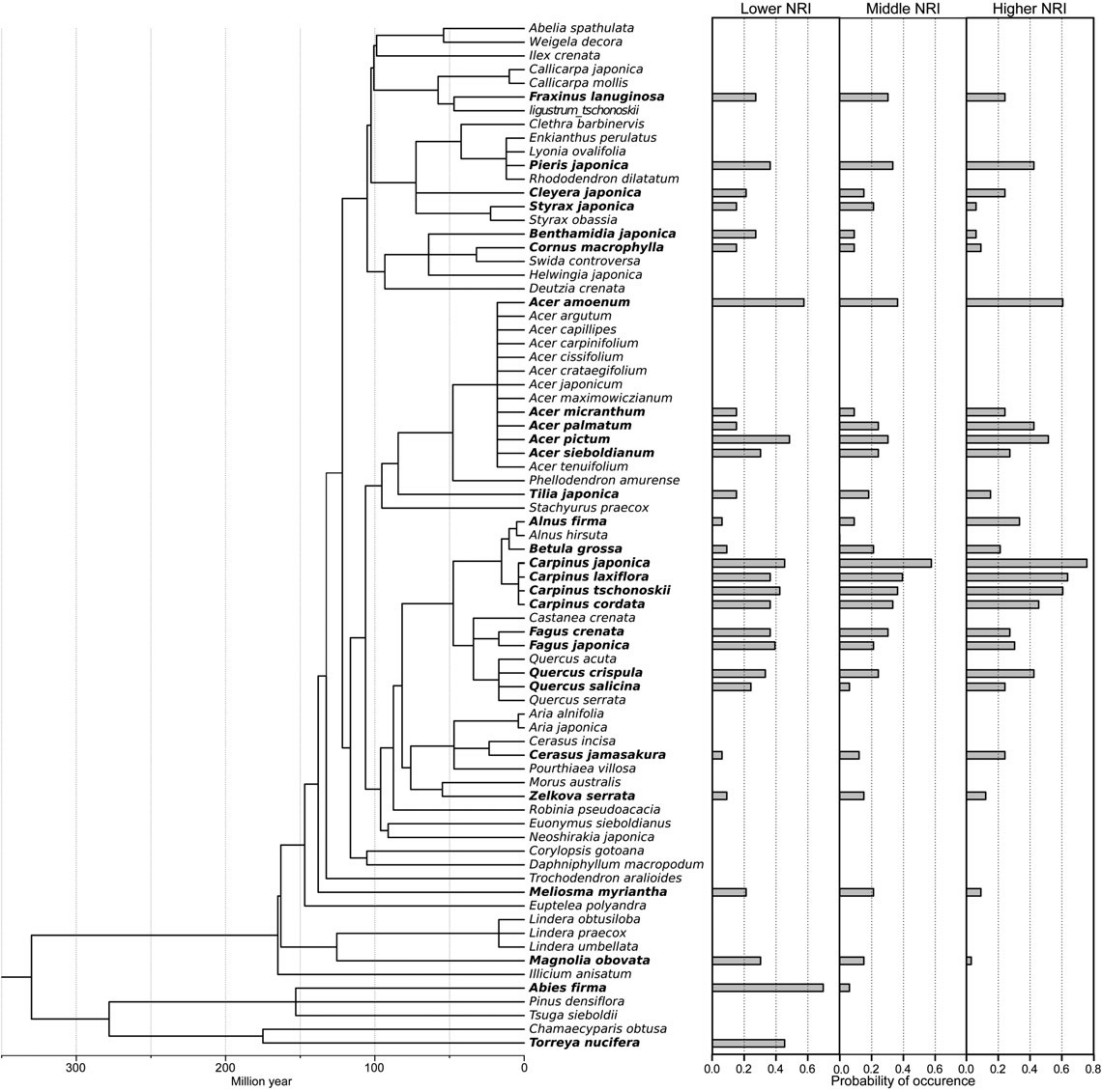
Phylogenetic tree for 75 woody plant species occurring in the 99 sampling plots. Bar charts on the right side show the occurrence ratio of 28 species that occurred in 10 or more plots. Plots are classified into three groups based on NRI for all species (see text). Each group includes 33 plots.

The 75 woody species that were non-climbing and had DBH ≥5 cm in the 306 ha watershed included 8 evergreen angiosperms, 62 deciduous angiosperms and 5 evergreen gymnosperms. These belonged to five monophyletic clades, eurosides (42 species), asteroids (20), magnolids (4), austrobaileyales (1), conifers (5) and other eudicots (3) (Fig. 2). Three *Carpinus* species, *Carpinus japonica*, *C. tschonoskii* and *C. laxiflora*, and *Acer amoenum* were frequent species in this catchment (they occurred in more than half of the 99 plots), whereas 21 species appeared in only one plot. The average number of species and individuals ± SD in a plot were 8.818 ± 3.102 and 17.263 ± 7.591, respectively.

The mean (±SD) per plot for NRI for all species, NTI for all species, NRI for angiosperms and NTI for angiosperms were 0.064 ± 1.108, 0.061 ± 1.108, 0.061 ± 1.018 and 0.031 ± 0.994, respectively. These variables varied with a unimodal distribution. They showed a significant negative correlation with Faith's PD; NRI for all species: *r*^2^ = 0.505, *P* < 0.001; NTI for all species: *r*^2^ = 0.498, *P* < 0.001; NRI for angiosperms: *r*^2^ = 0.198, *P* < 0.001 and NTI for angiosperms: *r*^2^ = 0.214, *P* < 0.001. However, they did not correlate with species richness; NRI for all species: *r*^2^ = 0.004, *P* = 0.947; NTI for all species: *r*^2^ = 0, *P* = 0.881; NRI for angiosperms: *r*^2^ = 0.016, *P* = 0.218 and NTI for all angiosperms: *r*^2^ = 0.016, *P* = 0.211. The number of plots that showed a significantly positive or negative deviation from a random assumption with a 95 % confidence level were as follows: five plots were positive and four plots were negative for NRI for all species, five plots each were positive and negative for NTI for all species, four plots were positive and two plots were negative for NRI for all angiosperms and three plots each were positive and negative for NTI for all angiosperms.

**Figure 3.**
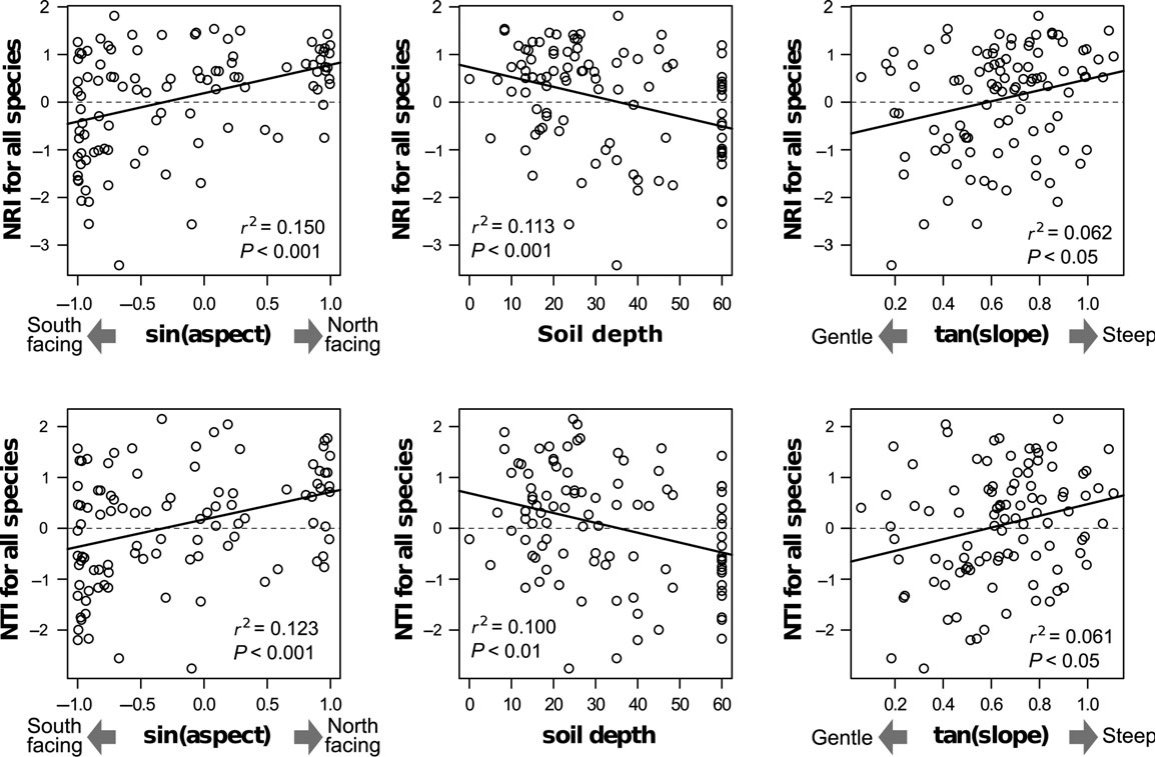
Single relationships between topographic variables selected in the best model and NRI and NTI for all species.

In the best models for all-species NRI and NTI, slope aspect, soil depth and slope inclination were selected (Table 1). These topographic variables were also selected in the other models with lower AIC values. Accordingly, NRI and NTI tended to be lower in plots with a gentle slope, a south-facing slope and with deep soil, and the values increased significantly for plots with a steep slope, a north-facing slope and with thin soil (Fig. 3). In addition to a similar topographical trend, elevation and lap8_3 were selected in the best model for NRI for all angiosperms, meaning that it was also higher at lower elevation and at more convex sites over small scales. For models of the NTI for all angiosperms, the AIC difference between the best and null model was small, and lower *r*^2^ values compared with other response variables were indicated. Although the phylogenetic trend was more non-explicit than the other three response variables, and soil depth was not selected, slope aspect and slope inclination as well as elevation and lap8_3 were selected for the best model.

**Table 1.** Top three significant and null generalized linear models for four response variables (NRI for all species, NTI for all species, NRI for angiosperm only and NTI for angiosperm only) of local wood communities in a temperate forest in a 306-ha water catchment, Mt Tanzawa, Japan. AIC, Akaike information criterion; tan(slope), tangent transformed slope inclination; lap8_3, lap8_19, convex/concavity in each spatial scale (see text).

Response variables	Models	Intercept	Standard multiple regression coefficients for selected explanatory variables						AIC	ΔAIC	*r*^2^
			Elevation	sin(aspect)	Soil depth	tan(slope)	lap8_3	lap8_19			
NRI for all species	1	0.064		0.396	−0.286	0.204			278.999	0.000	0.274
	2	0.064	−0.099	0.388	−0.280	0.182			279.980	0.981	0.282
	3	0.064		0.392	−0.286	0.208	0.050		280.723	1.723	0.276
	Null	0.064							304.293	25.294	–
NTI for all species	1	0.061		0.357	−0.265	0.206			283.732	0.000	0.237
	2	0.061	−0.123	0.347	−0.259	0.180			284.228	0.496	0.249
	3	0.061	−0.168	0.324	−0.251	0.197		0.144	284.314	0.583	0.264
	Null	0.061							304.206	20.474	–
NRI for angiosperms	1	0.061	−0.180	0.170	−0.176	0.236	0.145		273.857	0.000	0.212
	2	0.061	−0.171	0.182	−0.174	0.225			274.332	0.475	0.192
	3	0.061	−0.195		−0.193	0.233	0.159		275.231	1.374	0.186
	Null	0.061							287.477	13.620	–
NTI for angiosperms	1	0.031	−0.174	0.150		0.190	0.136		277.614	0.000	0.125
	2	0.031	−0.165	0.161		0.180			277.670	0.056	0.107
	3	0.031	−0.207	0.139		0.194		0.133	277.947	0.333	0.122
	Null	0.031							282.813	5.200	–

For the NRI for all species, Betulaceae including *Carpinus*, *Alnus* and *Betula*, *Acer palmatum*, *Acer micranthum* and *Cerasus jamasakura* tended to occur more frequently in plots with higher NRI (Fig. 2). In plots with significantly higher NRI, *C. tschonoskii* occurred in all five plots, *C. japonica* occurred in four plots and *C. laxiflora* occurred in three plots. The opposite tendency was observed for old-clade species, such as *Torreya nucifera*, *Abies firma* (conifers), *Magnolia obovata* and *Meliosma myriantha*. In addition, Fagus species, *Fagus crenata* and *F. japonica*, and *Benthamidia japonica* frequently occurred in the lower NRI plots compared with higher NRI plots (Fig. 2). For the significantly lower NRI plots, *T. nucifera* and *A. firma* occurred in all four plots, and *Tilia japonica* occurred in three plots. Moreover, it was noted that the species that tended to occur more frequently in plots with higher NRI also appeared in the lower NRI plots, although their occurrence ratio was different between lower and higher NRI plots.

## Discussion

### Deterministic processes in forest community structure on rugged terrain

We found that the phylogenetic community structure changed significantly along the topographic gradients. Namely, phylogenetically clustering was observed in plots on steep and north-facing slopes with thin soils, and a shift to phylogenetic over-dispersion towards gentle and south-facing slopes with deep soils. Even if conifers were removed, the NRI showed almost the same trend, although other topographic variables (elevation and lap8_3) were also effective. For the NTI without conifers, the phylogenetic trend along the topographic gradient was relatively non-explicit, and soil depth was not selected, unlike the best models for other response variables. This may depend on the effect of particular clades such as Betulaceae, Fagaceae and Acer (Aceraceae in the previous classification system before APGIII), which were frequent in this catchment. The NTI is more sensitive to ‘phylogenetic closeness’ within a community than the NRI. Some frequent clades often occurred simultaneously in the same plot in our data set. Thus, phylogenetic closeness within the community may be emphasized by species belonging to the same clades when conifers were removed. Then, the statistical power was reduced by the equalization of NTI values among communities. Although the NTI for all species showed almost the same trend as the NRI in this study, this should be interpreted carefully for the NTI calculated from low resolution trees such as in our study (Swenson 2009). Thus, discussion of this study was mainly based on the topographic trends of the NRI.

Although the effect of gymnosperms could not be ignored, we suggest that the phylogenetic trends along with topographic variables were obvious, and these phylogenetic trends were seemingly derived from the stress gradient that was related to the topography. A similar spectrum, i.e. a change in phylogenetic community structure coinciding with the intensity of abiotic stress, was reported in previous studies (Kembel and Hubbell 2006; Verdú and Pausas 2007; Ding *et al*. 2012; Spasojevic and Suding 2012); for example, Kembel and Hubbell (2006) reported phylogenetic clustering on plateaus suffering from seasonal drought and over-dispersion at sites with moist conditions on Barro Colorado Island in Panama. In addition, Spasojevic and Suding (2012) found the same phylogenetic trend along the abiotic stress gradient on alpine tundra.

There were steep and north-facing slopes with thin soils in the plots with relatively higher NRI for all species. Environmental conditions at these locations included not only insufficient light and poor nutrient levels but also ground instability. Landslide scars are frequently observed in this catchment, especially on steep slopes, where soils are easily eroded. Phylogenetic clustering in such highly disturbed sites was also reported (González-Caro *et al*. 2014). *Carpinus*, an abundant genus in this catchment (Kitagawa *et al*. 2014), was a characteristic clade for plots with relatively high NRI for all species. Such species are known to show a pioneer-like strategy that aims to enhance the chance of arrival and survival after or during frequent disturbances (Geritz *et al*. 1984). Their seedlings are predominantly shade intolerant, and they can dominate in gaps via abundant wind-dispersed seeds (Shibata and Nakashizuka 1995), namely a seed rain strategy (Alvarez-Buylla and Martínez-Ramos 1990). Among 28 major species analysed by Kitagawa *et al*. (2014), statistically significant habitat preferences for harsh sites were shown mainly for *Carpinus* species; biased distribution on steeper slopes was detected for *C. japonica* and *C. cordata* and also another Betulaceae, *Alnus firma*, a typical pioneer tree in this region. Biased distribution to plots with thinner soils was detected for *C. japonic*a and *C. laxiflora*. Moreover, individuals of *C. japonica* tended to grow to a large size on north-facing slopes (Kitagawa *et al*. 2014). Thus, we suggest that adaptive traits for the harsh topographic conditions may be conserved phylogenetically in the *Carpinus* clade, as well as their invasive ability as pioneers (Letcher 2010; Norden *et al*. 2012). As described in the Introduction, we expected that phylogenetically unique clades, such as *Euptelea polyandra*, would be distributed on such harsh sites disturbed frequently by active geomorphological processes (Sakai *et al*. 1995); however, such a trend was not detected. This was partly because minor species or species with limited distribution could not be identified sufficiently in analyses using this method. Nevertheless, it was interesting that a rather modern clade dominating the regional flora, *Carpinus*, can be considered to pass through abiotic environmental filters, bringing phylogenetic clustering to the community at the stressful end of the topographical gradient.

On the other hand, lower NRI values indicated that distantly related species tended to co-occur, i.e. phylogenetic over-dispersion at the opposite end of the topographic gradients. Environmental conditions in this location can be regarded as relatively mild. Such phylogenetic over-dispersion observed in relatively mild abiotic environmental conditions was often interpreted as a consequence of competition in previous studies (Kembel and Hubbell 2006; Spasojevic and Suding 2012). These studies suggested that many species establish owing to lower abiotic stress, but high competitive pressure among closely related species limited the phylogenetic similarity within the community and led to the coexistence of distantly related species. We considered that a similar mechanism also drives the lower values of NRI for all species in relatively mild conditions on the topographical gradients in this study. Kitagawa *et al*. (2014) analysed topographical trends in tree biomass in the same catchment and showed that total basal area of trees (Total BA) in a plot was higher on more gentle slopes. Here, the first causal factor of biomass, basal area of the maximum tree (MAX BA), showed the same trend, but the second factor, number of trees, did not. Although the NRI for all species was not significantly correlated with the number of trees, it was significantly negatively correlated with Total BA (*r*^2^ = 0.067, *P* < 0.01), MAX BA (*r*^2^ = 0.093, *P* < 0.01) and with mean basal area in a plot (*r*^2^ = 0.103, *P* < 0.01). This meant that the over-dispersion tendency observed in the relatively stress-free sites did not coincide with the abundance of competitors, but the existence of stronger competitors was effective. In addition, our previous study suggested that the spatial tendency of Total BA was largely contributed to by *F. crenata* and *A. firma* (Kitagawa *et al*. 2014), which were typical late-successional species in this region (Nozaki and Okutomi 1990). The occurrence ratios of two such species were relatively high in plots with lower NRI for all species (Fig. 2). Competition among trees is rather one sided; thus, late-successionals that could become larger trees are disproportionally much stronger (Kohyama and Takada 2009), and plots with lower NRI are considered to have higher competitive pressure. Therefore, we suggest that competitive exclusion may be the underlying mechanism for phylogenetic over-dispersion on the topographical gradients.

In addition to competitive exclusion, we also mention two other possible explanations for the phylogenetic over-dispersion, although the three explanations are not exclusive of each other. Firstly, we could not neglect the distribution of old-clade plants, because conifers obviously contributed to the lower NRI and higher PD. In fact, the significant negative correlation of the NRI for all species with Faith's PD suggested that the distribution pattern of old-clade species, such as conifers, affected the overall trend in PD, although the phylogenetic trend was sustained even if conifers were removed. Secondly, we suggest that lower NRI is associated with the late-successional stage. Studies in tropical forests found that changes in phylogenetic community structure from clustering to over-dispersion with the way of succession (Letcher 2010; Norden *et al*. 2012). They suggested that phylogenetic conservatism for pioneer-like strategies yielded phylogenetic clustering in early-successional communities, whereas phylogenetic over-dispersion was caused by a rise in species interaction in late-successional communities. This explanation can be adopted with our results. Thus, it can be said that topography governs PD via controlling the distribution of each species, successional status and thus the strength of competition in this rugged terrain. It is difficult to distinguish between the two explanations, successional stage or competition, because late-successional species were often stronger competitors within the forest community. However, we noted that local communities with different NRI values in this catchment could not be regarded as being in the same successional sequence. In other words, topographic gradient is not completely in accordance with successional gradient. Instead, there are rather specialized communities established in different environmental conditions depending on their topographic position (Sakai and Ohsawa 1994). Furthermore, the distribution pattern was different between the typical late-successional species (Kitagawa *et al*. 2014): *F. crenata* preferred stable ground with a small inclination, but *A. firma* had a biased distribution to south-facing slopes with deep soils. Thus, the late-successional communities were also a combination of different topo-communities. Although negative evidence for the implication of phylogenetic over-dispersion in the existing framework was alleged (e.g. Godoy *et al*. 2014), we suggest that biotic filtering may work as a dominant rule to achieve ‘phylogenetic over-dispersion’ in topographic conditions with lower abiotic stress in our study area.

### Stochastic processes in forest community structure on rugged terrain

Irrespective of the significant changes in phylogenetic community structure with topographical variables, as mentioned above, we found that the PD in most plots did not deviate statistically from the random expectation. This was in accordance with the fact that the species composition was not clearly differentiated among groups of plots with different values of NRI for all species. Notably, *Carpinus* was also a main group in high NRI plots, and *Fagus* appeared also in low NRI plots. In this rugged terrain with fine texture of steep relief, mutual stochastic invasions of early- and late-successional species may be enhanced because of the short distances required for conspicuous changes in environmental conditions. However, for *F. crenata*, which can grow to a large stature on deep soils (Kitagawa *et al*. 2014), invasion into areas with thin soils may be an opportunistic event and may not contribute its fitness. Such inconsistency between sites of occurrence and sites where species can grow to a large size was observed for all species that could be analysed in Kitagawa *et al*. (2014). Thus, if we could examine only mature individuals, a more deterministic pattern might be detected.

Nevertheless, disturbance is thought to be a major causal factor for stochastic processes. Because this region generally has highly erodible terrain, even plots that were in the most stress-free locations on the topographical gradients, the ground may be disturbed sometimes. Otherwise, even if the location is undisturbed, light may reach the forest floor from adjacent disturbed sites. Such conditions may enable early-successional species to immigrate and remain with late-successional competitors. Accordingly, we agree with general arguments that disturbances increase the chance of immigration by species with various niches from the regional species pool (Sousa 1984; Myers and Harms 2011), and stochastic immigration history could obscure the explicit community assembly rules (Chase 2010). However, we also noted that a stochastic process was not equal to random processes but was more or less regulated by the rules of the disturbance regime resulting from natural processes, such as geomorphological processes resulting in mountain denudation, as in the present case.

## Conclusions

In a mountainous forest area with steep and complex topography in Japan, we determined the assembly rules for the heterogeneous community structure of tree species. Community structure on the topographical gradient was basically controlled by deterministic processes including abiotic and biotic filtering. However, frequent surface disturbance due to unstable ground conditions increased the stochasticity and obscured the environment–community structure relations. These results were provided using the species characteristics reported by previous studies, and information on phylogenetic community structure. Thus, we suggest that the existing framework of the phylogenetic community approach is useful to understand the community assembly rules at a fine-grained vegetation structure level, at least in the present study area.

## Sources of Funding

This study was supported by a 2011 Joint Research Program C from the Research Institute of Environmental and Information Sciences, YNU.

## Contributions by the Authors

R.K. and A.S. originally formulated the idea; R.K., M.M. and A.S. developed original idea; R.K. conducted fieldwork; M.M., A.S.M. and A.S. jointed fieldwork; R.K., M.M. and A.S.M. performed statistical analyses and R.K., M.M., A.S.M. and A.S. wrote the manuscript.

## Conflict of Interest Statement

None declared.

## References

[PLV134C1] Alvarez-Buylla ER, Martínez-Ramos M 1990 Seed bank versus seed rain in the regeneration of a tropical pioneer tree. Oecologia 84:314–325. 10.1007/BF00329755 28313021

[PLV134C2] Cavender-Bares J, Ackerly DD, Baum DA, Bazzaz FA 2004 Phylogenetic overdispersion in Floridian oak communities. The American Naturalist 163:823–843. 10.1086/386375 15266381

[PLV134C3] Chang L, Chiu S, Yang K, Wang H, Hwong J, Hsieh C 2012 Changes of plant communities classification and species composition along the micro-topography at the Lienhuachih forest dynamics plot in the central. Taiwania 57:359–371.

[PLV134C4] Chase JM 2003 Community assembly: when should history matter? Oecologia 136:489–498. 10.1007/s00442-003-1311-7 12836009

[PLV134C5] Chase JM 2010 Stochastic community assembly causes higher biodiversity in more productive environments. Science 328:1388–1391. 10.1126/science.1187820 20508088

[PLV134C6] Clark DB, Clark DA, Read JM 1998 Edaphic variation and the mesoscale distribution of tree species in a neotropical rain forest. Journal of Ecology 86:101–112. 10.1046/j.1365-2745.1998.00238.x

[PLV134C7] De Oliveira AA, Vicentini A, Chave J, Castanho CDT, Davies SJ, Martini AMZ, Lima RAF, Ribeiro RR, Iribar A, Souza VC 2014 Habitat specialization and phylogenetic structure of tree species in a coastal Brazilian white-sand forest. Journal of Plant Ecology 7:134–144. 10.1093/jpe/rtt073

[PLV134C8] Ding Y, Zang R, Letcher SG, Liu S, He F 2012 Disturbance regime changes the trait distribution, phylogenetic structure and community assembly of tropical rain forests. Oikos 121:1263–1270. 10.1111/j.1600-0706.2011.19992.x

[PLV134C9] Duarte LS 2011 Phylogenetic habitat filtering influences forest nucleation in grasslands. Oikos 120:208–215. 10.1111/j.1600-0706.2010.18898.x

[PLV134C10] Enoki T 2003 Microtopography and distribution of canopy trees in a subtropical evergreen broad-leaved forest in the northern part of Okinawa Island, Japan. Ecological Research 18:103–113. 10.1046/j.1440-1703.2003.00549.x

[PLV134C11] Faith DP 1992 Conservation evaluation and phylogenetic diversity. Biological Conservation 61:1–10. 10.1016/0006-3207(92)91201-3

[PLV134C12] Fox J, Monette G 1992 Generalized collinearity diagnostics. Journal of the American Statistical Association 87:178–183. 10.1080/01621459.1992.10475190

[PLV134C13] Fukami T, Nakajima M 2011 Community assembly: alternative stable states or alternative transient states? Ecology Letters 14:973–984. 10.1111/j.1461-0248.2011.01663.x 21790934 PMC3187870

[PLV134C14] Geritz SAH, De Jong TJ, Klinkhamer PGL 1984 The efficacy of dispersal in relation to safe site area and seed production. Oecologia 62:219–221. 10.1007/BF00379016 28310716

[PLV134C15] Godoy O, Kraft NJB, Levine JM 2014 Phylogenetic relatedness and the determinants of competitive outcomes. Ecology Letters 17:836–844. 10.1111/ele.12289 24766326

[PLV134C16] González-Caro S, Umaña MN, Álvarez E, Stevenson PR, Swenson NG 2014 Phylogenetic alpha and beta diversity in tropical tree assemblages along regional-scale environmental gradients in northwest South America. Journal of Plant Ecology 7:145–153. 10.1093/jpe/rtt076

[PLV134C17] Gotelli NJ, Entsminger GL 2003 Swap algorithms in null model analysis. Ecology 84:532–535. 10.1890/0012-9658(2003)084[0532:SAINMA]2.0.CO;2 28547607

[PLV134C18] Harvey PH, Pagel MD 1991 The comparative method in evolutionary biology. Oxford: Oxford University Press.

[PLV134C19] Hillerislambers J, Adler PB, Harpole WS, Levine JM, Mayfield MM 2012 Rethinking community assembly through the lens of coexistence theory. Annual Review of Ecology, Evolution, and Systematics 43:227–248.

[PLV134C20] Hubbell SP 2001 The unified neutral theory of biodiversity and biogeography. Princeton: Princeton University Press. 10.1016/j.tree.2011.03.024 21561679

[PLV134C21] Hutchinson GE 1959 Homage to Santa Rosalia or why are there so many kinds of animals? The American Naturalist 93:145–159. 10.1086/282070

[PLV134C22] Ishikawa M, Hayashi S, Komito Y, Honda Y 2006 Slope failures triggered by the 1923 Kanto Earthquake and comparison with rainfall-induced slope failures. In: *American Geophysical Union, Fall Meeting 2006*, San Francisco, *abstract #S13C-0255*.

[PLV134C23] Kembel SW, Hubbell SP 2006 The phylogenetic structure of a neotropical forest tree community. Ecology 87:S86–S99. 10.1890/0012-9658(2006)87[86:TPSOAN]2.0.CO;2 16922305

[PLV134C24] Kembel SW, Cowan PD, Helmus MR, Cornwell WK, Morlon H, Ackerly DD, Blomberg SP, Webb CO 2010 Picante: R tools for integrating phylogenies and ecology. Bioinformatics 26:1463–1464. 10.1093/bioinformatics/btq166 20395285

[PLV134C25] Kitagawa R, Kondo H, Sakai A 2014 Spatial pattern of forest structure mediated by topography in a steep mountain basin in West Tanzawa, Japan. Journal of Forest Research 19:205–214. 10.1007/s10310-013-0406-1

[PLV134C26] Kohyama T, Takada T 2009 The stratification theory for plant coexistence promoted by one-sided competition. Journal of Ecology 97:463–471. 10.1111/j.1365-2745.2009.01490.x

[PLV134C27] Kraft NJB, Ackerly DD 2010 Functional trait and phylogenetic tests of community assembly across spatial scales in an Amazonian forest. Ecological Monographs 80:401–422. 10.1890/09-1672.1

[PLV134C28] Kraft NJB, Valencia R, Ackerly DD 2008 Functional traits and niche-based tree community assembly in an Amazonian forest. Science 322:580–582. 10.1126/science.1160662 18948539

[PLV134C29] Letcher SG 2010 Phylogenetic structure of angiosperm communities during tropical forest succession. Proceedings of the Royal Society B: Biological Sciences 277:97–104. 10.1098/rspb.2009.0865 PMC2842617 19801375

[PLV134C30] Losos JB 2008 Phylogenetic niche conservatism, phylogenetic signal and the relationship between phylogenetic relatedness and ecological similarity among species. Ecology Letters 11:995–1003. 10.1111/j.1461-0248.2008.01229.x 18673385

[PLV134C31] Magallón S, Hilu KW, Quandt D 2013 Land plant evolutionary timeline: gene effects are secondary to fossil constraints in relaxed clock estimation of age and substitution rates. American Journal of Botany 100:556–573. 10.3732/ajb.1200416 23445823

[PLV134C32] Masaki T, Suzuki W, Niiyama K, Iida S, Tanaka H, Nakashizuka T 1992 Community structure of a species-rich temperate forest, Ogawa Forest Reserve, central Japan. Vegetatio 98:97–111. 10.1007/BF00045549

[PLV134C33] Mayfield MM, Levine JM 2010 Opposing effects of competitive exclusion on the phylogenetic structure of communities. Ecology Letters 13:1085–1093. 10.1111/j.1461-0248.2010.01509.x 20576030

[PLV134C34] Mouquet N, Devictor V, Meynard CN, Munoz F, Bersier L-F, Chave J, Couteron P, Dalecky A, Fontaine C, Gravel D, Hardy OJ, Jabot F, Lavergne S, Leibold M, Mouillot D, Münkemüller T, Pavoine S, Prinzing A, Rodrigues ASL, Rohr RP, Thébault E, Thuiller W 2012 Ecophylogenetics: advances and perspectives. Biological Reviews 87:769–785. 10.1111/j.1469-185X.2012.00224.x 22432924

[PLV134C35] Myers JA, Harms KE 2011 Seed arrival and ecological filters interact to assemble high-diversity plant communities. Ecology 92:676–686. 10.1890/10-1001.1 21608476

[PLV134C36] Nagamatsu D, Miura O 1997 Soil disturbance regime in relation to micro-scale landforms and its effects on vegetation structure in a hilly area in Japan. Plant Ecology 133:191–200. 10.1023/A:1009743932202

[PLV134C37] Norden N, Letcher SG, Boukili V, Swenson NG, Chazdon R 2012 Demographic drivers of successional changes in phylogenetic structure across life-history stages in plant communities. Ecology 93:S70–S82. 10.1890/10-2179.1

[PLV134C38] Nozaki R, Okutomi K 1990 Geographical distribution and zonal interpretation of intermediate-temperate forests in eastern Japan. Japanese Journal of Ecology 40:57–69.

[PLV134C39] Pei N, Lian J-Y, Erickson DL, Swenson NG, Kress WJ, Ye W-H, Ge X-J 2011 Exploring tree-habitat associations in a Chinese subtropical forest plot using a molecular phylogeny generated from DNA barcode loci. PLoS ONE 6:e21273 10.1371/journal.pone.0021273 21701680 PMC3119057

[PLV134C40] Qian H, Ricklefs RE 2000 Large-scale processes and the Asian bias in species diversity of temperate plants. Nature 407:180–182. 10.1038/35025052 11001054

[PLV134C41] Qian H, Ricklefs RE, White PS 2005 Beta diversity of angiosperms in temperate floras of eastern Asia and eastern North America. Ecology Letters 8:15–22. 10.1111/j.1461-0248.2004.00682.x

[PLV134C42] Qian H, Hao Z, Zhang J 2014 Phylogenetic structure and phylogenetic diversity of angiosperm assemblages in forests along an elevational gradient in Changbaishan, China. Journal of Plant Ecology 7:154–165. 10.1093/jpe/rtt072

[PLV134C43] Sakai A, Ohsawa M 1994 Topographical pattern of the forest vegetation on a river basin in a warm-temperate hilly region, central Japan. Ecological Research 9:269–280. 10.1007/BF02348413

[PLV134C44] Sakai A, Ohsawa T, Ohsawa M 1995 Adaptive significance of sprouting of *Euptelea polyandra*, a deciduous tree growing on steep slopes with shallow soil. Journal of Plant Research 108:377–386. 10.1007/BF02344363

[PLV134C45] Shibata M, Nakashizuka T 1995 Seed and seedling demography of four co-occurring *Carpinus* species in a temperate deciduous forest. Ecology 76:1099–1108. 10.2307/1940918

[PLV134C46] Sousa WP 1984 The role of disturbance in natural communities. Annual Review of Ecology and Systematics 15:353–391. 10.1146/annurev.es.15.110184.002033

[PLV134C47] Spasojevic MJ, Suding KN 2012 Inferring community assembly mechanisms from functional diversity patterns: the importance of multiple assembly processes. Journal of Ecology 100:652–661. 10.1111/j.1365-2745.2011.01945.x

[PLV134C48] Swenson NG 2009 Phylogenetic resolution and quantifying the phylogenetic diversity and dispersion of communities. PLoS ONE 4:e4390 10.1371/journal.pone.0004390 19194509 PMC2633039

[PLV134C49] Swenson NG, Enquist BJ 2009 Opposing assembly mechanisms in a neotropical dry forest: implications for phylogenetic and functional community ecology. Ecology 90:2161–2170. 10.1890/08-1025.1 19739378

[PLV134C50] Verdú M, Pausas JG 2007 Fire drives phylogenetic clustering in Mediterranean Basin woody plant communities. Journal of Ecology 95:1316–1323. 10.1111/j.1365-2745.2007.01300.x

[PLV134C51] Verdú M, Rey PJ, Alcántara JM, Siles G, Valiente-Banuet A 2009 Phylogenetic signatures of facilitation and competition in successional communities. Journal of Ecology 97:1171–1180. 10.1111/j.1365-2745.2009.01565.x

[PLV134C52] Webb CO 2000 Exploring the phylogenetic structure of ecological communities: an example for rain forest trees. The American Naturalist 156:145–155. 10.1086/303378 10856198

[PLV134C53] Webb CO, Ackerly DD, Mcpeek MA, Donoghue MJ 2002 Phylogenies and community ecology. Annual Review of Ecology and Systematics 33:475–505. 10.1146/annurev.ecolsys.33.010802.150448

[PLV134C54] Webb CO, Ackerly DD, Kembel SW 2008 Phylocom: software for the analysis of phylogenetic community structure and trait evolution. Bioinformatics 24:2098–2100. 10.1093/bioinformatics/btn358 18678590

[PLV134C55] Wikström N, Savolainen V, Chase MW 2001 Evolution of the angiosperms: calibrating the family tree. Proceedings of the Royal Society B: Biological Sciences 268:2211–2220. 10.1098/rspb.2001.1782 PMC1088868 11674868

